# Temporomandibular Disorders Diagnosis: Current Challenges and the Promising Role of Artificial Intelligence

**DOI:** 10.1055/s-0046-1816081

**Published:** 2026-02-16

**Authors:** Tahani Mohammed Binaljadm, Redhwan Saleh Al-Gabri, Samah Saker, Hanan Omar AboAlrejal, Musab Hamed Saeed, Ahmed Yaseen Alqutaibi

**Affiliations:** 1 Department of Substitutive Dental Sciences (Prosthodontics), College of Dentistry, Taibah University, Al Madinah, Saudi Arabia; 2 Department of Prosthodontics, Faculty of Dentistry, Ibb University, Ibb, Yemen; 3 Department of Prosthodontics, Faculty of Dentistry, National University, Ibb, Yemen; 4 Department of Oral and Maxillofacial Radiology, Faculty of Dentistry, Ibb University, Ibb, Yemen; 5 Department of Clinical Science, College of Dentistry, Ajman University, Ajman City, United Arab Emirates

**Keywords:** temporomandibular disorders, diagnosis, artificial intelligence, deep learning, DC/TMD, imaging analysis, predictive modeling

## Abstract

Temporomandibular disorders (TMDs) are a group of musculoskeletal and joint-related conditions affecting the temporomandibular joint (TMJ), masticatory muscles, and associated structures. They are among the most common causes of non-dental orofacial pain and functional impairment, significantly affecting quality of life. Despite advances in assessment and the development of standardized diagnostic systems such as the Research Diagnostic Criteria (RDC/TMD) and Diagnostic Criteria for Temporomandibular Disorders (DC/TMD), accurate diagnosis remains difficult due to the multifactorial nature of TMDs, variability in symptoms, and subjectivity in pain reporting. Diagnostic accuracy is further limited by interexaminer variability, symptom overlap with other orofacial pain conditions, and restricted access to advanced imaging techniques. Artificial intelligence (AI) has emerged as a promising approach to address these challenges. Machine learning and deep learning algorithms can process complex imaging, clinical, and psychosocial data to improve diagnostic accuracy, consistency, and efficiency. AI-assisted imaging has shown strong performance in detecting disc displacement, degenerative changes, and other TMJ abnormalities, while predictive models based on symptoms, wearable sensors, and AI-driven decision-support tools are broadening diagnostic capabilities. This review summarizes current challenges in TMD diagnosis and highlights the growing role of AI in this field. Integrating AI technologies with established frameworks such as the DC/TMD may enable more objective, data-driven, and personalized diagnostic approaches. Ongoing interdisciplinary research, clinical validation, and ethical implementation are crucial for realizing AI's potential to transform TMD diagnosis and enhance patient outcomes.

## Introduction

Temporomandibular disorders (TMDs) encompass a heterogeneous group of musculoskeletal and joint-related conditions affecting the temporomandibular joint (TMJ), masticatory muscles, and associated structures. They are among the most prevalent causes of non-dental orofacial pain and functional limitation, second only to chronic low back pain among musculoskeletal disorders. 1 2 3 Epidemiological data suggest that TMDs affect approximately 5 to 12% of the population, with a higher prevalence in females during the second to fourth decades of life. 4 5 6 7 8 9 Chronic TMDs, defined by pain persisting for more than 3 months, are associated with significant impairment in mastication, speech, and psychological well-being, thereby reducing overall quality of life. 3 4 10

Accurate and early diagnosis is critical for optimizing management outcomes and preventing chronicity. However, achieving diagnostic precision remains a challenge due to the multifactorial nature of TMDs and the subjectivity of symptom reporting. 11 12 13 14 15 16 17 Clinically, diagnosis relies primarily on detailed patient history, physical examination, and assessment of functional patterns such as jaw movement and joint sounds. 1 2 18 Imaging modalities—including panoramic radiography, computed tomography (CT), cone-beam computed tomography (CBCT), and magnetic resonance imaging (MRI)—serve as adjunctive tools for confirming intra-articular pathology or degenerative changes. 7 11 19 20 21 22 23 Despite their diagnostic utility, imaging results may not always correlate with symptom severity, and interpretation often requires specialized expertise. 24 25 26

To improve diagnostic reliability and facilitate research comparability, standardized systems such as the DC/TMD were developed. 14 27 28 29 These frameworks introduced a dual-axis model that integrates both clinical and psychosocial dimensions of the disorder. Nevertheless, limitations persist—including inter-examiner variability, overlap with other orofacial pain conditions, and limited access to advanced imaging—highlighting the need for more objective and reproducible diagnostic methods. 11 12 13 14 15

In recent years, artificial intelligence (AI) has emerged as a transformative tool capable of addressing these diagnostic gaps. By leveraging machine learning, deep learning, and predictive analytics, AI systems can process complex multimodal data—including imaging, clinical, and behavioral inputs—to enhance diagnostic precision, reproducibility, and efficiency. AI applications in TMDs diagnosis include automated image analysis, symptom-based predictive modeling, wearable sensor integration, and AI-enhanced screening tools. 30 31 32 33 34 35 36 37 38 39

The purpose of this review was to synthesize current evidence on conventional TMDs diagnostic methods, elucidate their inherent challenges, and explore the evolving role of AI in enhancing diagnostic accuracy and accessibility. By integrating current clinical knowledge with emerging computational advances, this review aimed to provide a comprehensive understanding of how AI technologies may reshape the diagnostic landscape of TMDs. Although several reviews have summarized TMD diagnosis, the present review is positioned as a narrative, translational synthesis. Instead of cataloging studies, we focus on current status and how AI can be integrated into clinical frameworks, clinician workflows, and telehealth systems, emphasizing practical implications for real-world implementation.

## Evolution of TMD Diagnostic Criteria

### Research Diagnostic Criteria (RDC/TMD) and Diagnostic Criteria (DC/TMD) for Temporomandibular Disorders

Given their multifactorial etiology involving biological, psychological, and social factors, TMDs present considerable diagnostic complexity. To minimize variability and enable standardized assessment, structured systems such as the RDC/TMD and its successor, DC/TMD, were developed. 1 14 40 Comparisons of the RDC/TMD and DC/TMD based on Sójka et al 41 are presented in Table 1 .

**Table 1:** Comparison of the RDC/TMD and DC/TMD based on Sójka et al 41

Aspect	RDC/TMD (1992)	DC/TMD (2014)
Development background	Introduced by Dworkin and LeResche (1992) 40 as the first standardized system for TMD research	Developed by Schiffman et al. (2014) 14 as an updated, evidence-based revision improving validity and reliability
Conceptual model	Primarily biomedical; focused on physical and psychological assessment	Multidimensional biopsychosocial model emphasizing pain, psychosocial, and behavioral factors
Structure	Dual-axis system: • Axis I—Clinical/radiographic diagnosis • Axis II—Psychological status and pain-related disability	Dual-axis system (retained but refined): • Axis I—Validated clinical diagnostic criteria • Axis II—Simplified, standardized psychosocial assessment tools
Axis I—Diagnostic groups	Three groups (8 subdiagnoses): I. Muscle disorders (myofascial pain ± limited opening) II. Disc displacements (with/without reduction) III. Arthralgia, osteoarthritis, osteoarthrosis	Expanded taxonomy (up to 12 subdiagnoses): – Myalgia (local, myofascial, with referral) – Arthralgia – Headache attributed to TMD (HATMD) – Disc displacements (including intermittent locking) – Degenerative joint disease, subluxation
Axis I—Diagnostic validity	Moderate validity; reliable algorithms but limited accuracy (especially for joint disorders)	High validity and reliability (e.g., sensitivity ≥ 0.86, specificity ≥ 0.98 for common pain-related TMDs)
Axis II—Psychological assessment	Included Graded Chronic Pain Scale (GCPS), depression (SCL-90 subscales), and nonspecific physical symptoms	Simplified and expanded tools: GCPS v2.0, PHQ-9 (depression), GAD-7 (anxiety), PHQ-15 (physical symptoms), Oral Behaviors Checklist, Jaw Functional Limitation Scale
New diagnostic categories	None beyond core groups	Added new categories: headache attributed to TMD (HATMD), subluxation, disc displacement with reduction, and intermittent locking. Removed: myofascial pain with limited opening
Clinical examination	Detailed but complex; required imaging for differentiation	Simplified, standardized clinical protocol with examiner commands, symptom questionnaire, and diagnostic decision trees
Screening tools	No specific screening questions	Introduced validated tools like the TMD Pain Screener and 3Q/TMD screening questions
Evidence base	Primarily research-based; not fully validated for clinical application	Evidence-based and validated for both clinical and research use, allows diagnostic standardization globally
Focus of improvement	Foundation for structured TMD research	Emphasis on clinical practicality, diagnostic accuracy, and global standardization
Overall strength	Reliable but limited validity and clinical applicability	High validity, simplified procedures, new diagnostic entities, and better psychosocial integration

#### Evolution from RDC/TMD to DC/TMD

The RDC/TMD, introduced in 1992, represented a pivotal step toward establishing an evidence-based diagnostic framework for TMDs. 2 3 It was the first protocol to employ a dual-axis system—Axis I, addressing clinical conditions of the TMJ and masticatory muscles, and Axis II, evaluating psychosocial status and pain-related disability. The RDC/TMD provided consistent definitions for myofascial pain, disc displacement, and arthralgia, enabling systematic investigation of TMDs etiology and prevalence across populations. 3

Over time, clinical experience and empirical research revealed several limitations of the RDC/TMD, including variability in diagnostic reliability and insufficient inclusion of emerging psychosocial parameters. Consequently, the DC/TMD was introduced in 2014 as an enhanced version, offering greater validity, simplified clinical application, and updated psychometric properties. 2 It preserved the two-axis model but refined both diagnostic algorithms and assessment tools. Axis I now includes a comprehensive classification of specific disorders—such as myalgia, myofascial pain with referral, arthralgia, degenerative joint disease, and subluxation—while Axis II employs validated instruments for assessing depression, anxiety, and somatization, aligned with the biopsychosocial model of pain. 3 18 42

#### Structure and Diagnostic Framework

Both systems adopt a multidimensional diagnostic structure:

- **Axis I (clinical assessment)** : Focuses on physical diagnoses based on clinical and imaging findings. The DC/TMD introduced standardized clinical examination protocols and clarified diagnostic criteria for muscle-related and joint-related disorders.
- **Axis II (psychosocial assessment)** : Evaluates psychological distress, pain-related disability, and behavioral factors using validated self-report questionnaires such as the Patient Health Questionnaire (PHQ-9, PHQ-15) and Generalized Anxiety Disorder scale (GAD-7). 19 20 43 44 45 46 These instruments quantify emotional and behavioral aspects contributing to TMD chronicity and severity.

The DC/TMD further allows integration with additional validated tools, including the Oral Behavior Checklist (OBC) for parafunctional habits, 24 43 47 the Pittsburgh Sleep Quality Index (PSQI) for sleep disturbances, 21 27 43 48 and the Oral Health Impact Profile (OHIP) for assessing quality of life. 28 43 Together, these measures support comprehensive patient evaluation consistent with modern pain medicine principles.

#### Clinical and Research Significance

The adoption of RDC/TMD and DC/TMD has profoundly influenced both clinical diagnostics and research methodology. Their use enables the standardization of case definitions, facilitating cross-study comparison and meta-analytic synthesis. In systematic analyses employing these criteria, TMD was consistently associated with psychological variables—notably depression, anxiety, somatization, and sleep disturbance—alongside biological and anatomical factors such as female sex, oral parafunction, and condylar asymmetry. 4 19 20 21 29 43 44 47 48 49 50 51 These findings reinforce the biopsychosocial model, underscoring the interdependence between psychological distress, behavioral responses, and musculoskeletal pathology. 5 18 42 52

The RDC/TMD was instrumental in demonstrating the link between psychological factors and chronic pain, while the DC/TMD refined this integration by providing more reliable instruments for assessing mental health and behavioral risk factors. For instance, studies utilizing the DC/TMD framework confirmed significant associations between TMDs symptoms and poor sleep quality, high anxiety levels, and depressive symptoms. 19 21 43 48 Similarly, research using the RDC/TMD found that somatization and oral parafunctions such as clenching and grinding strongly contribute to TMD pain. 20 44 47 53

#### The Biopsychosocial Foundation

The transition from RDC/TMD to DC/TMD mirrors the evolution of pain theory from a purely biomedical to a biopsychosocial perspective. This model recognizes that biological dysfunction, psychological distress, and social environment collectively shape the clinical presentation of chronic conditions like TMD. 52 The DC/TMD's integration of psychological screening tools aligns with this philosophy, allowing clinicians to identify and manage contributing emotional and behavioral dimensions early.

Studies employing these diagnostic criteria have shown that women exhibit a higher prevalence of TMD, likely influenced by hormonal and psychosocial mechanisms. 19 20 21 29 43 47 50 54 55 56 57 Psychological comorbidities—especially depression and anxiety—are consistently linked to increased pain perception and disability, suggesting that early psychological assessment is vital for effective management. 19 20 29 44 58 59 60 Likewise, parafunctional habits and sleep disturbances exacerbate muscular strain and pain sensitivity, reinforcing the need for a multidisciplinary approach. 4 5 56

#### Contemporary Implications

Currently, the DC/TMD serves as the global standard for both clinical diagnosis and research on TMDs. Its validated structure ensures diagnostic consistency across populations and fosters interdisciplinary collaboration among dentists, psychologists, and medical specialists. The RDC/TMD remains valuable historically and in comparative studies, but the DC/TMD offers superior reliability, psychometric strength, and clinical usability. 2 3

By incorporating structured clinical evaluation with psychosocial screening, the DC/TMD allows clinicians to tailor interventions that address both the physical and psychological components of TMDs. This integrative framework has redefined diagnostic accuracy, improved treatment outcomes, and strengthened the evidence base for TMD management within a biopsychosocial context.

### Other Diagnostic Approaches

#### Clinical Evaluation

The diagnosis of TMDs primarily relies on a thorough patient history and detailed clinical examination. Characteristically, symptoms are closely associated with jaw function—such as pain or discomfort during mouth opening, closing, or chewing—and are often localized to the preauricular, temporal, or masseteric regions. If pain remains unaffected by mandibular movements, clinicians should consider other potential sources of orofacial pain. Audible joint sounds, including clicking, popping, or grating, are frequently reported in TMDs; however, such noises can also be present in nearly half of asymptomatic individuals. 61

A large retrospective analysis encompassing 4,528 cases over 25 years identified facial pain (96%), ear discomfort (82%), headache (79%), and jaw dysfunction (75%) as the most prevalent presenting symptoms. 62 Other, less specific manifestations may include dizziness, neck or back pain, and even ocular or upper limb discomfort. Chronic TMDs are defined as when symptoms, particularly pain, persist for more than 3 months 10 ( Fig. 1 ).

**Fig. 1:**
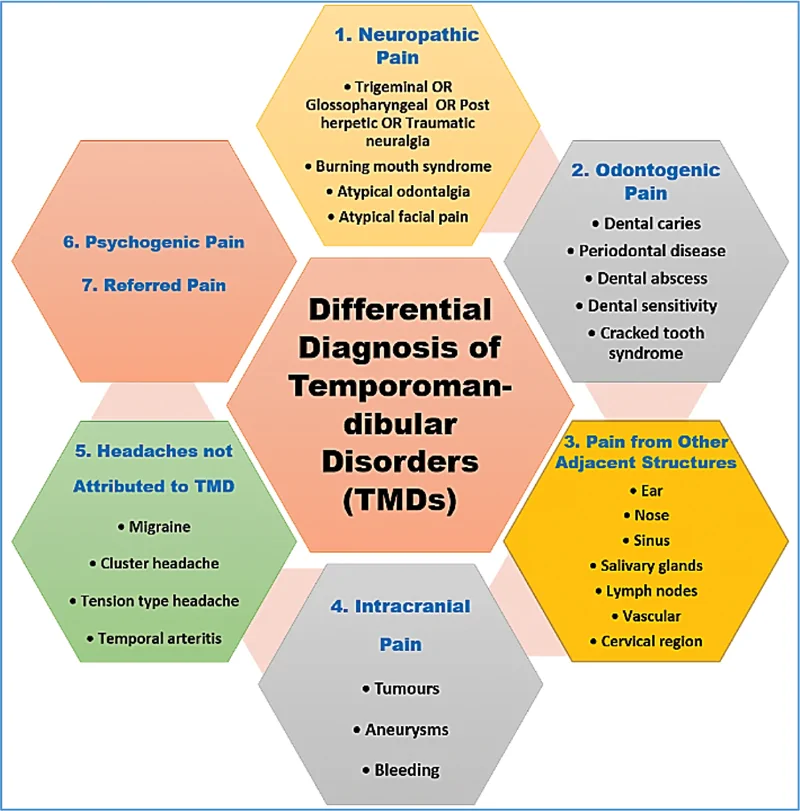
Differential diagnosis of temporomandibular disorders (TMDs). 64

#### Physical Examination Findings

Physical findings that support a TMD diagnosis include restricted mandibular motion, deviation or deflection during opening, tenderness over masticatory muscles, pain with dynamic loading, and palpable joint abnormalities. Signs of bruxism and tenderness in cervical or shoulder muscles are also common. Clinicians should evaluate for dental or skeletal malocclusions—such as edentulism, occlusal asymmetries, or poorly adapted restorations—that may contribute to TMDs development. Importantly, cranial nerve deficits should not be attributed to TMDs and warrant further neurological evaluation. 6 63

Joint sounds may provide diagnostic clues. A single click during opening suggests anterior disc displacement, while a second click on closing indicates recapture of the displaced disc, known as disc displacement with reduction. In contrast, when disc displacement prevents normal condylar translation and limits mouth opening, a “closed lock” state is present. Crepitus, on the other hand, often signifies degenerative articular changes consistent with osteoarthritis. 64

Tenderness elicited upon palpation of the TMJ is indicative of intra-articular pathology, whereas myalgia or referred pain can be identified through tenderness in the masseter, temporalis, or cervical muscles. Deviation of the mandible toward the symptomatic side during opening is frequently associated with anterior disc displacement. 22

#### Imaging Modalities

Imaging serves as an adjunct when clinical assessment yields inconclusive findings. 65 Several modalities are available, each offering distinct diagnostic value. The initial evaluation often includes plain or panoramic radiography, which effectively identifies gross abnormalities such as fractures, dislocations, or advanced degenerative changes. CT provides superior delineation of subtle osseous morphology compared with standard radiographs. 11 23

MRI remains the gold standard for comprehensive TMJ evaluation, particularly for assessing soft-tissue structures and internal derangements. The correlation between MRI findings and clinical joint pathology ranges from 78 to 95% in symptomatic individuals, 7 8 22 66 although up to one-third of asymptomatic patients may demonstrate false-positive findings. 67 Therefore, MRI is generally reserved for cases with persistent symptoms, failed conservative management, or suspected intra-articular derangement.

When MRI is unavailable or contraindicated, ultrasonography offers a practical, dynamic, and cost-effective alternative for assessing internal derangements. 68 Despite its operator dependence, it can provide valuable real-time information about joint function and disc position.

#### Diagnostic Injections

Local anesthetic injections targeting the muscles of mastication or auriculotemporal nerve region can serve as a diagnostic adjunct, particularly for differentiating myogenous from arthrogenous sources of pain. Such procedures should only be conducted by trained clinicians familiar with TMJ anatomy to minimize complications. A lack of pain relief following appropriate nerve blockade suggests an alternative etiology and warrants reevaluation of the diagnosis. 69

## Challenges in Conventional TMD Diagnosis

Despite the availability of standardized diagnostic frameworks such as the RDC/TMD and the DC/TMD, clinical diagnosis remains problematic due to the multifactorial nature of TMDs, variability in clinical presentation, and subjective patient reporting. Several key barriers contribute to these diagnostic difficulties ( Fig. 2 ). 5 12 13 14 15 16 25 26 70

**Fig. 2:**
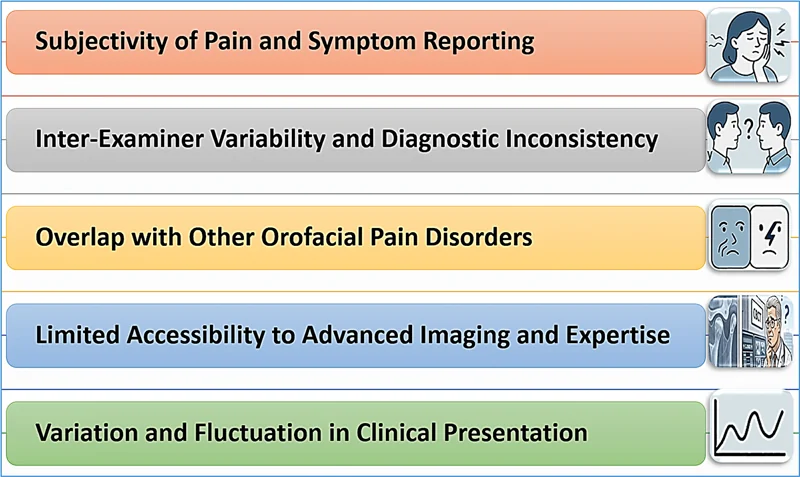
Challenges in conventional temporomandibular disorders (TMDs) diagnosis.

### Subjectivity of Pain and Symptom Reporting

Pain, the primary presenting symptom in TMD, is highly subjective and influenced by psychosocial and biological factors. Patients often struggle to describe their pain precisely, using nonspecific descriptors such as “tightness,” “ache,” or “pressure,” which can obscure the underlying cause and hinder accurate clinical interpretation. Approximately 65% of individuals with TMDs experience fluctuating or cyclic pain, alternating between periods of exacerbation and remission, further complicating diagnostic assessment. 12 71 72

Moreover, psychosocial conditions such as anxiety, depression, and chronic stress influence pain perception, exacerbating the subjective component of TMDs symptomatology. 17 73 74 75 The biopsychosocial model underscores the need to integrate psychological evaluation within diagnostic protocols to account for these complexities. 18 42 60 76

### Interexaminer Variability and Diagnostic Inconsistency

Clinical examination for TMDs heavily relies on the examiner's palpation technique, interpretation of joint sounds, and evaluation of jaw movement, all of which are prone to variability. Even with structured systems such as RDC/TMD and DC/TMD, inconsistencies in diagnostic judgment persist due to differing clinician experience and interpretation thresholds. The lack of universally accepted clinical markers and overlap of findings between muscle-related and joint-related TMDs further complicate classification. 14 77 78 As reported by Schiffman et al, 14 diagnostic inconsistency remains a major limitation in both clinical and research applications, emphasizing the need for objective diagnostic methods such as imaging-based or AI-assisted assessments.

### Overlap with Other Orofacial Pain Disorders

A significant diagnostic challenge arises from the symptom overlap between TMD and other orofacial pain conditions. Disorders such as odontogenic pain, sinusitis, salivary gland diseases, headache syndromes, and cervicogenic pain can mimic TMDs in presentation. 15 16 For example, a throbbing ache in the maxillary area may stem from sinus or dental pathology rather than TMJ dysfunction. Additionally, TMDs-related pain often radiates beyond the joint region—up to 85% of patients report referred pain to the ear, temple, or forehead, and 25% describe headaches rather than jaw pain. 5 13 This broad referral pattern, coupled with overlapping pain pathways, often leads to misdiagnosis or delayed treatment. Therefore, multidisciplinary collaboration among dentists, neurologists, and pain specialists is critical for accurate differential diagnosis.

### Limited Accessibility to Advanced Imaging and Expertise

While advanced imaging modalities such as MRI and CBCT can reveal structural changes in the TMJ and surrounding tissues, their diagnostic utility in TMDs is constrained by accessibility, cost, and interpretive expertise. Furthermore, imaging results can be inconclusive—radiographic abnormalities may be present in asymptomatic individuals, whereas patients with clear clinical TMDs symptoms may show normal imaging findings. These discrepancies undermine clinicians' confidence in imaging as a definitive diagnostic tool. The limited availability of trained orofacial radiologists further compounds this issue, especially in nonspecialized clinical settings. 25 26 70

### Variation and Fluctuation in Clinical Presentation

TMDs present with diverse clinical manifestations involving the joint, masticatory muscles, or both. Some patients experience unilateral symptoms, while others have bilateral or alternating presentations. Pain intensity and localization can vary over time, often influenced by stress, occlusal factors, or systemic health conditions. This fluctuation in clinical expression complicates longitudinal assessment and standardization of diagnostic outcomes. 5

## Applications and Key Benefits of AI in TMDs Diagnosis

TMDs diagnosis is often challenging due to overlapping symptoms, multifactorial etiology, and the subjective nature of pain. AI has emerged as a transformative tool in addressing these diagnostic complexities, providing enhanced precision, efficiency, and reproducibility through advanced data analytics and pattern recognition. AI applications in TMDs diagnosis span imaging-based analysis, symptom-based predictive modeling, wearable technology integration, and the development of AI-driven screening tools ( Fig. 3 ).

**Fig. 3:**
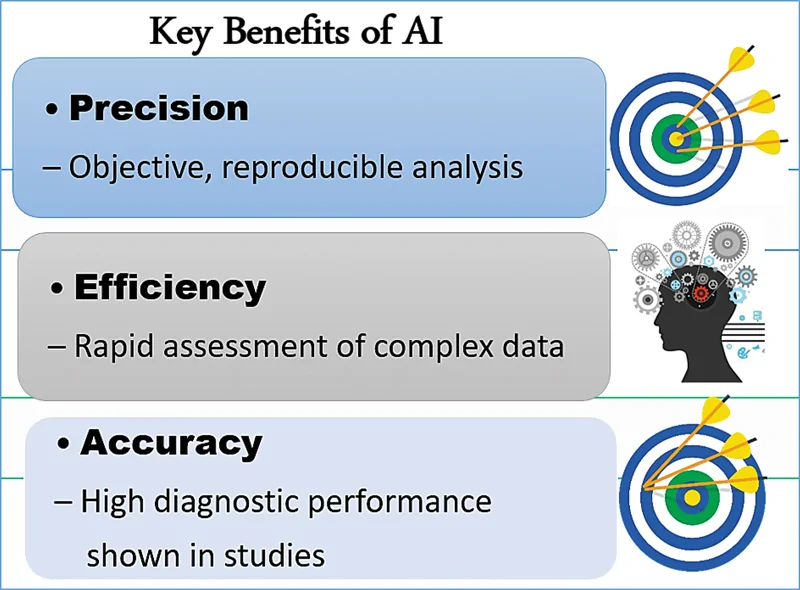
Transforming temporomandibular disorders (TMDs) diagnosis with artificial intelligence (AI).

### Imaging-Based AI Models

AI has demonstrated substantial potential in automating the interpretation of TMJ imaging, including CBCT, MRI, panoramic radiography, and infrared thermography (IT). Deep learning architectures—particularly convolutional neural networks (CNNs)—enable automated segmentation, feature extraction, and classification of osseous and soft-tissue structures, offering an objective and reproducible complement to human interpretation.

AI-assisted analysis of MRI and CBCT has improved the detection of disc displacement, condylar morphology alterations, and osteoarthritic changes. Reported diagnostic accuracies for TMJ imaging models range from 59 to 100%, with sensitivity between 0.76 and 0.80 and specificity from 0.63 to 0.95. 30 Early efforts, such as the web-based neural network by de Dumast et al (2018), achieved 91% diagnostic agreement with experts by integrating 3D CBCT data with clinical and biological parameters for TMJ osteoarthritis. 79 Similarly, Shoukri et al demonstrated that coupling CBCT-derived condylar features with salivary and serum biomarkers improved diagnostic accuracy to 91.2%, underscoring the value of multimodal AI models that merge morphological and biochemical data. 80 Recent advancements in CNN architectures have further enhanced diagnostic precision. Eşer et al used the YOLOv5 model to classify TMJ osteoarthritis on CBCT sagittal images, achieving an area under the curve (AUC) of 0.97 and 76.8% accuracy for identifying erosive and osteophytic changes. 81

Likewise, Sankar et al showed that MobileNetV2 and ResNet achieved superior sensitivity (0.735–1.0) and accuracy (>83%) for TMJ disc position detection, outperforming traditional approaches. 31 Comparable performance was achieved by Kar et al, who reported 92.4% accuracy and an AUC of 0.95 using a CBCT-based CNN. 33 MRI-based studies have explored more sophisticated multistage frameworks. Kao et al combined U-Net for joint segmentation with CNN classifiers (InceptionV3, DenseNet169) for disc displacement detection, achieving up to 85% accuracy. 82 Min et al enhanced diagnostic interpretability through a hybrid DeepLabV3+ and random forest model, yielding 89% accuracy for anterior disc displacement—superior to single-stage CNNs. 83 Similarly, Li et al used transfer-learning with ResNet152 and super-resolution MRI to identify anterior disc displacement, achieving 76.8% accuracy (AUC: 0.834) and outperforming clinicians. 84 Lee et al reported 74.2% accuracy and 87.3% specificity for detecting TMJ effusion using ensemble CNN models, demonstrating the utility of visualization tools such as Grad-CAM for model interpretability. 85

Hybrid CNN–transformer frameworks have also emerged as promising tools. Ozsari et al achieved up to 97% accuracy (AUC = 0.95) for identifying disc displacement and condylar degeneration using models such as MobileNetV2, ResNet-101, and Xception. 86 Complementarily, Kim et al applied multilayer perceptron and random forest algorithms to MRI data for predicting disc perforation, with the MLP model reaching an AUC of 0.94, outperforming conventional MRI interpretation. 87

Beyond conventional modalities, lightweight architectures such as YOLO and GoogleNet have shown reliable detection of osteoarthritic and degenerative joint changes, occasionally exceeding human performance. 32 34 Non-radiographic imaging, including infrared thermography analyzed through MLP algorithms, also demonstrated high precision and sensitivity for TMDs detection. 38 Collectively, these studies highlight AI's growing capacity for rapid, objective, and reproducible imaging-based assessment of TMJ pathology. While high diagnostic accuracy has been achieved across multiple architectures and modalities, challenges remain regarding model generalizability, dataset heterogeneity, and the integration of imaging with clinical or molecular data to improve real-world applicability.

### Symptom- and Data-Based Predictive Models

Beyond imaging, AI has been increasingly applied to analyze multifactorial clinical data encompassing pain patterns, functional limitations, and psychosocial variables. Machine learning algorithms can classify symptom patterns, integrate behavioral data, and predict disease subtypes or chronicity.

Lee et al validated the myTMJ mobile diagnostic tool, which achieved a 95.5% diagnostic accuracy—surpassing that of orofacial pain specialists (90%). 36 Reda et al combined symptom-based diagnostic data with natural language processing, enhancing diagnostic accuracy among nonspecialist dentists. 37 Other models utilizing Bagging and Multivariate Adaptive Regression Splines (MARS) achieved up to 89.7% accuracy and an AUC of 0.939 in predicting TMD presence from clinical variables. 39

Large-scale studies, such as that by Lee et al, demonstrated that random forest models could identify critical TMDs risk factors—including stress, BMI, and socioeconomic status—emphasizing AI's value in integrating multifactorial data for more holistic diagnostic insights. 35

### Wearable Devices and Sensor-Based AI

Wearable and sensor-based technologies are expanding the frontier of AI-assisted diagnosis through continuous and objective data collection. Jaw kinematic trackers and electromyography (EMG) sensors provide real-time information on mandibular movement patterns and muscle activity, which can be analyzed using AI algorithms to identify abnormal functional signatures associated with TMDs.

These systems can detect subtle deviations in motion trajectories, asymmetries in muscle activation, and excessive parafunctional activity, offering early diagnostic potential and personalized monitoring. For instance, AI-integrated occlusal splints embedded with stress sensors have shown high precision in occlusal force detection and feedback for bruxism-related TMDs. 88 Such innovations signify a shift toward continuous, data-driven diagnostics beyond the clinical setting.

### AI-Enhanced Screening and Decision-Support Tools

AI-powered screening tools and mobile applications are increasingly being developed for teledentistry and chairside use. These tools combine questionnaire data, image input, and predictive modeling to assist clinicians and patients in preliminary assessment and triage.

Decision-support systems such as myTMJ exemplify this trend by integrating multimodal data for self-guided diagnostic screening. 36 Additionally, conversational AI platforms like ChatGPT-4 have demonstrated moderate to high reliability and content quality when providing TMD-related information, enhancing both clinician education and patient awareness. 89 Recent work evaluating TMD-related content on social media platforms has shown that while online engagement is high, the quality and reliability of information vary substantially, reinforcing the importance of developing evidence-based AI-driven educational and screening tools. 90

Incorporating AI into the DC/TMD workflow can be conceptualized across three complementary roles. First, AI-based prescreening systems, such as myTMJ and symptom-based ML models, support early recognition and referral by nonspecialists. 36 37 Second, AI serves most effectively as an auxiliary diagnostic support during DC/TMD evaluation, where CNN- and YOLO-based imaging models for TMJ osteoarthritis and disc displacement improve objectivity and reduce interexaminer variability. 32 34 81 86 91 Third, postdiagnostic predictive models integrating clinical and psychosocial variables show high accuracy in identifying risk and chronicity but require multicenter longitudinal validation before guiding personalized care. 39 92 Collectively, AI as an augmented decision-support layer within DC/TMD appears to be the most readily accepted and clinically feasible pathway.

## Future Perspectives

The integration of AI into TMDs diagnosis represents a promising frontier in dental and orofacial pain research. While current AI applications—particularly those employing deep learning and machine learning algorithms—have demonstrated notable accuracy in imaging-based detection of disc displacement, osteoarthritic changes, and morphological abnormalities, 30 31 33 34 82 86 the field remains in its developmental phase. Future research should focus on multimodal data integration, combining imaging findings, clinical symptoms, psychosocial variables, and biological markers to generate more comprehensive and personalized diagnostic profiles. Such multidimensional AI models could align closely with the biopsychosocial model central to the DC/TMD framework. 49 50

Despite the promise of multimodal AI systems, integrating heterogeneous data presents several challenges. These include dimensional imbalance between high-resolution imaging data and lower-dimensional clinical or psychosocial inputs, as well as difficulties aligning features that reflect fundamentally different biological or behavioral constructs. 93 94 Temporal asynchrony between imaging, symptoms, and psychosocial assessments, along with frequent missing data, further complicates model development. 95 Multimodal fusion also requires interpretable architectures to ensure clinical transparency. 94 96 Early studies suggest imaging features primarily drive structural diagnosis, while psychosocial variables support chronicity and outcome prediction; however, quantitative comparisons remain scarce, reinforcing the need for large, standardized multimodal datasets. 95 97

Another key direction involves improving model generalizability and validation. Many current studies employ limited datasets or single-center samples, which restrict external validity. Emerging evidence suggests that some AI models, particularly those trained on restricted or homogeneous datasets, may show reduced accuracy when tested across populations differing in craniofacial morphology, gender distribution, or ethnic background. This raises concerns regarding algorithmic bias and reinforces the need for large, demographically diverse multicenter datasets to ensure equitable diagnostic performance. 98 99 Collaborative multicenter initiatives and open-access data repositories are necessary to train and validate AI models across diverse populations, imaging modalities, and diagnostic protocols. 30 31 33 Furthermore, standardization of performance metrics—including sensitivity, specificity, and AUC—would enhance comparability across studies and facilitate regulatory evaluation.

The development of explainable AI (XAI) systems is equally important to ensure clinician trust and transparency. Black-box models, while powerful, can obscure the rationale behind diagnostic outputs. Explainable frameworks could provide visual or statistical justification for AI-based conclusions, enabling practitioners to verify and interpret algorithmic reasoning alongside clinical judgment.

Integration of AI tools into clinical workflows and telehealth platforms presents another promising avenue. Mobile diagnostic tools such as myTMJ and AI-enhanced decision-support systems have already shown high accuracy and feasibility for remote or preliminary screening. 36 89 100 Incorporating these tools into electronic health records and teledentistry platforms could expand access to expert-level diagnostic support, especially in resource-limited or rural settings ( Fig. 4 ).

**Fig. 4:**
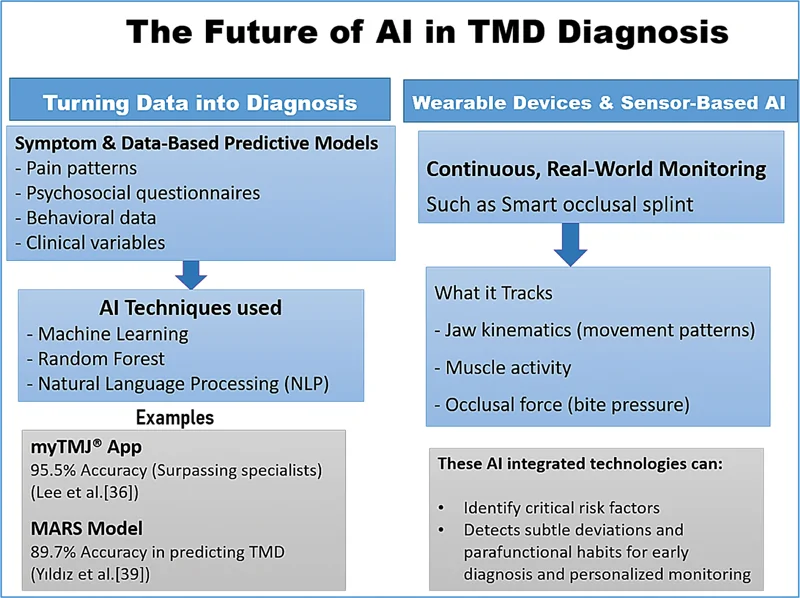
AI beyond the scan: the emerging role of predictive models and wearable sensor-based technologies in TMDs diagnosis and monitoring.

Finally, ethical, educational, and regulatory considerations must guide AI adoption in clinical practice. Issues of patient data privacy, algorithmic bias, and the need for clinician training in AI literacy remain critical. Developing standardized clinical guidelines for AI validation, interpretation, and integration will be essential to ensure responsible and equitable use. As AI continues to evolve, interdisciplinary collaboration among clinicians, computer scientists, psychologists, and policymakers will be vital to translate technological advances into meaningful clinical benefit.

## Conclusion

TMDs remain diagnostically challenging due to their multifactorial nature, overlapping symptoms, and strong psychosocial influences. Although frameworks such as the RDC/TMD and DC/TMD have improved diagnostic structure and consistency, limitations related to subjectivity, interexaminer variability, and restricted imaging access persist. AI offers a promising avenue to address these gaps: imaging-based deep learning models now achieve high accuracy in identifying structural abnormalities, while predictive algorithms, wearable technologies, and AI-driven decision-support tools enhance early detection and integrate clinical, behavioral, and psychosocial data.

However, successful translation into practice requires overcoming key barriers—including multimodal data integration, algorithmic bias, and limited external validation of current models. Moving forward, the synergistic incorporation of AI within the DC/TMD diagnostic framework, supported by multicenter datasets, XAI, and ethical implementation, holds the potential to transform TMD assessment into a more objective, reproducible, and patient-centered process.
